# Access to firearms: essential factor for risk management in psychiatry

**DOI:** 10.1192/bjo.2021.352

**Published:** 2021-06-18

**Authors:** Donnchadh Walsh, Una Fallon, Sonn Patel, Elizabeth Walsh

**Affiliations:** 1 School of Medicine, University College Dublin; 2 Galway Menal Health Service; 3 University Hospital Galway

## Abstract

**Objective:**

Increase awareness of the risk associated with access to firearms in clinical practice.

**Case report:**

A 57-year-old man, with a 30 year history of schizophrenia, was reviewed routinely at home. His illness is predominantly characterised by chronic delusions of a grandiose nature. He believes he has been offered various senior employment positions and has acted on these beliefs by presenting at workplaces in business attire. He has no insight into his condition. At review, he described awakening a week earlier in a panic and seizing hold of his legally held shotgun. He planned to shoot out the window as he believed people were breaking in. His wife prevented him from doing so by taking the gun and hiding it. A few days later he found the gun and intended to frighten off potential pursuers by pretending to shoot birds. He was persuaded to surrender the gun and it was taken to the local Garda (Police). A short time later, he presented to Garda Headquarters, over an hour away, seeking the return of his gun. At review, he had limited insight into the potential seriousness of the situation. The team immediately liaised with Gardaí, a HCR- 20 risk assessment was completed and clozapine levels checked.

**Discussion:**

We had not know that our patient owned a shotgun despite very regular contact with him. During a comprehensive psychiatric history we routinely ask about risk of harm to self and others, but rarely ask specifically about access to or ownership of guns. Working on a farm, rural living or having an interest in shooting sports may raise the issue. Suicide, security breaches and homicide are the main risks conferred by firearms in mental illness. Mental illness is not necessarily prohibitive to gun ownership. Applicants for gun certificates in the UK must disclose specific medical conditions, including a psychotic illness, and an automatic medical report is sought. In the Irish Republic it is the responsibility of the applicant to declare any specific physical or mental health condition. Although a medical report may be sought, it is not automatic in all cases. Lack of insight into psychotic illness may potentially influence self-declaration upon application for a certificate.

**Conclusion:**

Awareness of a persons access to firearms should be part of our routine risk assessment.

